# Efficacy and safety of hypoglycemic drugs in improving cognitive function in patients with Alzheimer's disease and mild cognitive impairment: A systematic review and network meta-analysis

**DOI:** 10.3389/fneur.2022.1018027

**Published:** 2022-11-30

**Authors:** Xin-Chen Wang, Chen-Liang Chu, Han-Cheng Li, Kuan Lu, Cheng-Jiang Liu, Ye-Feng Cai, Shi-Jian Quan, Shi-Jie Zhang

**Affiliations:** ^1^Department of Pharmaceutical Engineering, College of Food and Pharmaceutical Engineering, Zhaoqing University, Zhaoqing, China; ^2^Department of General Medicine, Affiliated Anqing First People's Hospital of Anhui Medical University, Anqing, China; ^3^Department of Neurology, The Second Affiliated Hospital of Guangzhou University of Chinese Medicine, Guangzhou, China; ^4^School of Pharmaceutical Sciences, Guangzhou University of Chinese Medicine, Guangzhou, China

**Keywords:** hypoglycemic drugs, cognitive function, Alzheimer's disease, mild cognitive impairment, a systematic review, network meta-analysis

## Abstract

**Objective:**

The purpose of this study was to compare the effects of oral hypoglycaemic drugs (HDs) on cognitive function and biomarkers of mild cognitive impairment (MCI) and Alzheimer's disease (AD) through a network meta-analysis of randomized controlled trials (RCTs).

**Methods:**

We conducted systematic searches for English- and Chinese-language articles in the PubMed, Medline, Embase, Cochrane Library and Google Scholar databases, with no date restrictions. We performed a network meta-analysis, which we report here according to the Preferred Reporting Items for Systematic Reviews and Meta-Analyses (PRISMA). The 16 studies included a total of 3,081 patients. We selected the Mini-Mental State Examination (MMSE), the Alzheimer's Disease Assessment Scale-Cognitive section (ADAS-Cog), the Alzheimer's Disease Cooperative Study Activities of Daily Living section (ADCS-ADL) and amyloid beta (Aβ) 42 as the outcome measures for analysis and comparison.

**Result:**

We selected seven treatments and assessed the clinical trials in which they were tested against a placebo control. Of these treatments, intranasal insulin 20 IU (ITSN20), glucagon-like peptide-1 (GLP-1), and dipeptidyl peptidase 4 inhibitor (DPP-4) were associated with significantly improved MMSE scores (7 RCTs, 333 patients, 30≥MMSE score≥20: mild) compared with placebo [standardized mean difference (SMD) 1.11, 95% confidence interval (CI) (0.87, 1.35); SMD 0.75, 95% CI (0.04, 1.41); and SMD 4.08, 95% CI (3.39, 4.77), respectively]. Rosiglitazone 4 mg (RLZ4), rosiglitazone 10 mg (RLZ10), intranasal insulin 40 IU (ITSN40), and ITSN20 significantly decreased ADAS-Cog scores (11 RCTs, 4044 patients, 10 ≤ ADAS-Cog scores ≤ 30: mild and moderate) compared with placebo [SMD −1.40, 95% CI (−2.57, −0.23), SMD −3.02, 95% CI (−4.17, −1.86), SMD −0.92, 95% CI (−1.77, −0.08), SMD −1.88, 95% CI (−3.09, −0.66)]. Additionally, ITSN20 and ITSN40 significantly improved ADCS-ADL scores (2 RCTs, 208 patients, ADCS-ADL scale score ≤ 10: mild) compared with placebo [SMD 0.02, 95% CI (0.01, 0.03), and SMD 0.04, 95% CI (0.03, 0.05), respectively]. In the 16 included studies, the degree of AD was classified as mild or moderate. For mild cognitive impairment, DPP-4 performed best, but for mild to moderate impairment, ITSN40 had excellent performance.

**Conclusion:**

Various HDs can improve the cognitive function of MCI and AD patients. Different drug regimens brought different degrees of improvement, which may be related to their dosage, duration, and mechanism of action.

**Systematic review registration:**

www.crd.york.ac.uk/prospero.

## Introduction

Alzheimer's disease (AD) is a type of neurodegenerative dementia characterized by an impaired ability to encode and store new memories in the early stage, followed by the gradual degradation of cognition and behaviour to the point of clinical manifestations (1–3). As currently understood, the mechanism of AD dementia consists mainly of the cleavage of amyloid precursor protein (APP), the deposition of amyloid beta (Aβ), and the accumulation of hyperphosphorylated tau protein. The above factors lead to decreased synaptic strength and neurodegeneration (4, 5). Mild cognitive impairment (MCI) is an abnormal mental condition between normal cognitive status and dementia. The main clinical manifestations of MCI are a decline in cognitive function, a deficit in episodic memory, and a decline in the ability to carry out complex daily activities. Although they do not meet the diagnostic criteria for dementia, elderly patients with MCI are a high-risk group for AD (6, 7).

Diabetes is one of the most prevalent metabolic diseases in the world, according to statistics reported by the World Health Organization, the number of diabetes cases worldwide is expected to reach 366 million by 2030(8, 9). A large amount of epidemiological evidence supports an association between diabetes and MCI and AD. The cognitive decline rate of diabetes patients is twice the rate associated with normal aging, and the diabetes patients also have an elevated risk of MCI (10). A meta-analysis found that the relative risk of MCI in patients with diabetes compared to those without was 1.21 (11). Biessels et al. (12) showed that the risk of AD in diabetes patients was almost twice that of nondiabetic patients of the same age. Poor blood glucose control and a long duration of diabetes are risk factors for AD (13). Indeed, two large-scale national population studies with a follow-up time of approximately 10 years have confirmed that these features are risk factors (14).

Although numerous studies have been carried out to develop drugs for the treatment of MCI and AD, no effective cure has been found; current treatments can only reduce symptoms and delay disease progression. Therefore, it is necessary to research and develop drugs with stronger efficacy and novel mechanisms of action (15). The hypothesis that hypoglycaemic drugs (HDs) can improve MCI and AD has been widely considered. It has been found that intranasal insulin (ITSN), metformin (MTN), pioglitazone (PLZ), rosiglitazone (RLZ), glucagon-like peptide-1 (GLP-1) and dipeptidyl peptidase 4 inhibitor (DPP-4) agonists can improve the metabolism and nutrition of synapses, nerves and glia, alleviate neuroinflammatory reactions, and regulate memory and other cognitive and emotional functions, mainly due to insulin sensitization and other direct effects independent of the insulin signalling mechanism of the above drugs (16).

To study the potential use of HD in the treatment of MCI and AD, we selected ITSN, MTN, PLZ, RLZ, GLP-1 and DPP-4 as intervention measures, including ITSN 20 IU (ITSN20) and ITSN 40 IU (ITSN40), RLZ 2 mg (RLZ2), RLZ 4 mg (RLZ4), RLZ 8 mg (RLZ8), and RLZ 10 mg (RLZ10), and reviewed relevant randomized clinical trials to evaluate the efficacy of HDs in patients with AD and MCI by network meta-analysis (NMA).

## Materials and methods

### Registration

The study protocol was registered with the International Prospective Register of Systematic Reviews (PROSPERO) under the following registration number: CRD42022355924.

### Search strategy and data extraction

We strictly adhered to the PRISMA (Preferred Reporting Items for Systematic Reviews and Meta-Analyses) reporting guidelines (17, 18). The databases Public Medicine (PubMed), Medline, Excerpta Medica Database (Embase), Cochrane Library and Google Scholar were searched as of June 2022. The following MESH terms were applied to search for relevant literature: “Hypoglycemic drugs” OR “Hypoglycemic Agent” OR “Hypoglycemic Agent” OR “Antihyperglycemic Agent” OR “Antidiabetic Drug” OR “Intranasal insulin” OR “Metformin” OR “Dimethylbiguanidine” OR “Glucophage” OR “Metformin” OR “Hydrochloride” OR “Metformin HCl” OR “Pioglitazone” OR “Pioglitazone Hydrochloride” OR “Rosiglitazone” OR “Rosiglitazone Maleate” OR “Glucagon like peptide GLP-1 receptor” OR “DPP-4” OR “Dipeptidyl-Peptidase IV Inhibitors” OR “DPP-4 Inhibitor”, and “Alzheimer's Disease”, “Mild Cognitive Impairment”.

In addition, we reviewed numerous references from the retrieved articles and sought out other literature materials, such as research reports and conference reports. The search scope was limited to randomized controlled trials (RCTs) in humans. The reference lists of included articles were reviewed, and relevant studies were sought as comprehensively as possible to avoid omissions. Working independently, the two reviewers (XC and CL) reviewed the titles and abstracts, summarized the search results, and applied the inclusion and exclusion criteria. XC and CL used the Cochrane Guidelines (19) to assess the risk of bias and the quality of the included trials. If there was a disagreement between the two reviewers, the third author (SJ) made the final decision.

### Study selection

We followed the PICOS (population, interventions, comparisons outcomes, study designs) when defining the eligibility criteria. The studies included in the review met the following conditions: (1) the patients met their country's diagnostic standard for MCI or AD, (2) the intervention measure was an HD, and the control was a placebo, (3) the outcome measures included the Mini-Mental State Examination (MMSE), the Alzheimer's Disease Assessment Scale Cognitive subscale (ADAS-Cog), and/or the Alzheimer's Disease Cooperative Study Activities of Daily Living subscale (ADCS-ADL), and (4) the study was an RCT.

The exclusion criteria were as follows: (1) the subjects were not human (2) the article was a review or an *in vitro* cell experiment, (3) HDs were used in the control group, (4) cognitive function outcome indicators were not included, and (5) the data were incomplete.

### Outcome measures

In contrast to a traditional meta-analysis, our NMA does not extract the relevant results of each study separately but rather extracts, combines, and analyses results from across RCTs. The outcome measures included the MMSE to measure intelligence and the degree of cognitive impairment, the ADAS-Cog score to detect the level of cognitive ability, and the ADCS-ADL scale score to detect the ability to perform activities of daily living.

### Statistical analysis

We applied Stata 17.0 software to the extracted continuous variables for NMA and generated the standardized mean difference (SMD) with its 95% confidence interval (CI) or the odds ratio (OR) with its 95% CI. The statistical heterogeneity criteria for the application of the fixed-effects model were I^2 < ^ 50%, *p* > 0.01. If these criteria were not met, the random-effects model was used. Publication bias and small-sample effects were assessed by funnel plots. Each result was ranked using the surface under the cumulative ranking curve (SUCRA). The higher the SUCRA value, the better the curative effect that may be achieved. A matrix was developed to compare all interventions and detect whether the SUCRA difference between each pair of interventions reached a significant level. The consistency or inconsistency of these relationships was evaluated to enhance the stability of the results. The threshold for statistical significance was *p* < 0.05. Subgroup analysis by treatment duration was completed with Review Manager 5.3 software.

## Results

### Literature search and included studies

**Figure 1:**
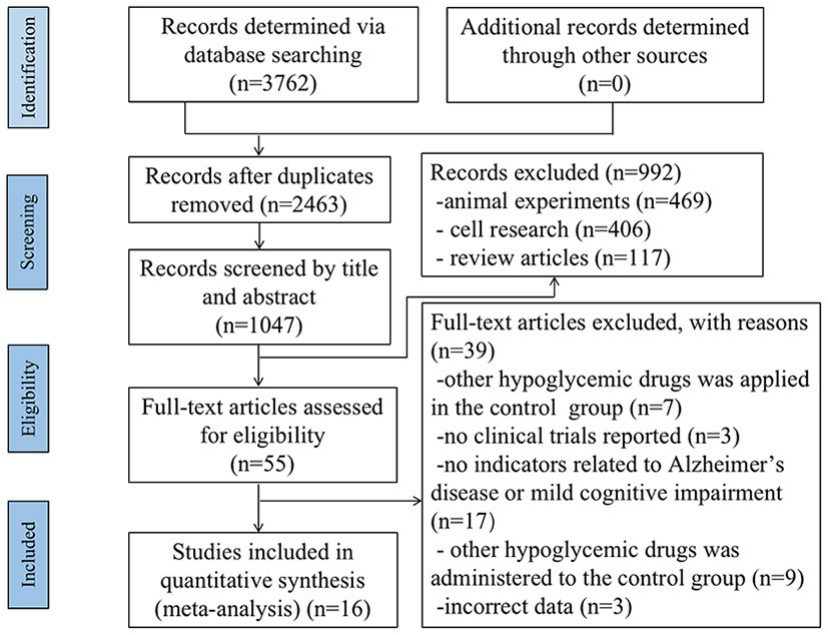
PRISMA flow diagram for search and selection of eligible studies included in the network meta-analysis.

A total of 3,762 studies were selected from the five databases according to the search strategy. After the duplicate articles were removed and the titles and abstracts were screened, 55 studies remained; these studies were then evaluated in full-text form. Thirty-nine studies were excluded based on the full text. Ultimately, there were 16 eligible studies that included 3081 patients meeting the inclusion criteria for the NMA; 2870 patients were diagnosed with AD, and 211 patients were diagnosed with MCI (Figure 1). Information on the included studies is listed in Table 1.

**Table 1:** The information about the included studies.

**References**	**Randomized sample size:I/C**	**Intervention (/once)**	**Control**	**Treatment duration (Month)**	**Cognitive assessment**
Suzanne Craft (20)	36, 38/30	20 IU of Insulin 40 IU of Insulin	Placebo	48 M	ADAS-Cog score ADCS-ADL scale score
Suzanne Craft (21)	12/12	20 IU of Insulin	Placebo	4 M	MMSE score
Maja Mustapica (22)	33, 32/26	20 IU of Insulin 40 IU of Insulin	Placebo	4 M	ADAS-Cog score
Amy Claxtona (23)	36, 38/30	20 IU of Insulin 40 IU of Insulin	Placebo	4 M	ADAS-Cog score ADCS-ADL scale score
José A. Luchsinger (24)	40/40	500 mg Metformin	Placebo	32 M	MMSE score ADAS-Cog score
Michael Rosenbloom (25)	19/16	20 IU of Insulin	Placebo	6 M	ADAS-Cog score
David S. Geldmacher (26)	15/14	15 mg Pioglitazone	Placebo	18 M	ADAS-Cog score
Haruo Hanyu (27)	15/17	15 mg−30 mg Pioglitazone	Placebo	6 M	MMSE score
TomohikoSato (28)	21/21	15 mg−30 mg Pioglitazone	Placebo	6 M	MMSE score ADAS-Cog score
Risner (29)	127,130/122	2 mg Rosiglitazone 4 mg Rosiglitazone 8 mg Rosiglitazone	Placebo	6 M	ADAS-Cog score
C. Harrington (30)	473, 459/461	2 mg Rosiglitazone 8 mg Rosiglitazone	Placebo	12 M	ADAS-Cog score
Michael Gold (31)	162, 156/159	2 mg Rosiglitazone 8 mg Rosiglitazone	Placebo	6 M	ADAS-Cog score
Michael C. Irizarrya (32)	733/856	Rosiglitazone	Placebo	6 M	ADAS-Cog score
Qiang Li (33)	24/21	0.6 mg−1.8 mg GLP-1	Placebo	3 M	MMSE score
Roger J. Mullins (34)	11/10	GLP-1	Placebo	18 M	MMSE score ADAS-cog score
Jujun Xue (35)	30/30	100 mg Sitagliptin	Placebo	6 M	MMSE score

I/C, Intervention/Control; ADAS-Cog, Alzheimer's Disease Assessment Scale-Cognitive section; ADCS-ADL, Alzheimer's Disease Cooperative Study-Activities of Daily Living Scale; MMSE, Mini-mental State Examination; GLP-1Glucagon-like Peptide-1.

### Pairwise meta-analysis

**Figure 2:**
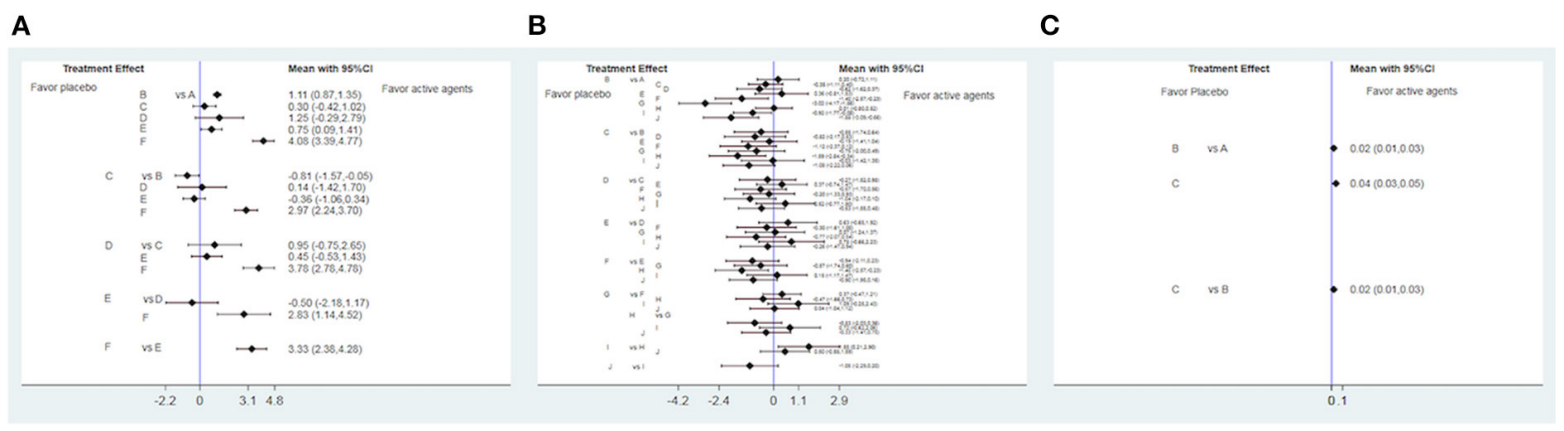
Forest plot efficacy of hypoglycemic drugs (HD) with placebo **(A)** Forest plot efficacy of HD with placebo in improving MMSE score. A: placebo, B: intranasal insulin, C: metformin, D: pioglitazone, E: glucagon-like peptide-1 (GLP-1), F: dipeptidyl peptidase 4 inhibitor (DPP-4). **(B)** Forest plot efficacy of HD with placebo in improving ADAS-Cog score. A: placebo, B: metformin, C: pioglitazone, D: glucagon-like peptide-1, E: rosiglitazone 2 mg, F: rosiglitazone 4 mg, G: rosiglitazone 10 mg, H: rosiglitazone 8 mg, I: intranasal insulin 20 IU, J: intranasal insulin 40 IU. **(C)** Forest plot efficacy of HD with placebo in improving ADCS-ADL scale score. A: placebo, B: intranasal insulin 20 IU, C: intranasal insulin 40 IU.

**Figure 3:**
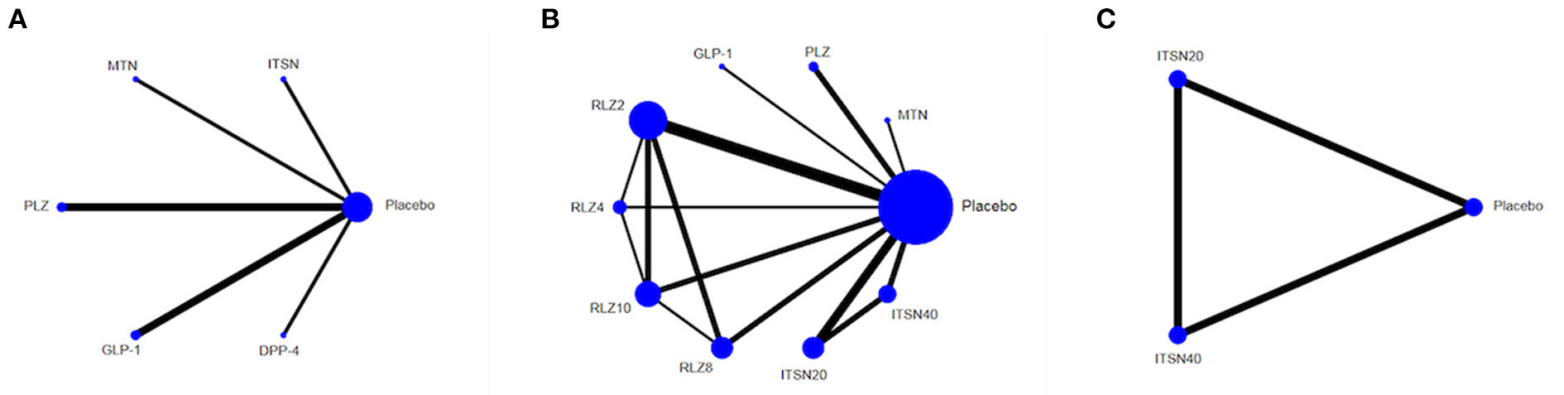
Network map for hypoglycemic drugs (HD) with placebo in improving MMSE score. **(A)** ADAS-Cog score, **(B)** ADCS-ADL scale score, **(C)** Lines connect the interventions that have been studied in head-to-head comparisons in eligible RCTs. The width of the lines represents the total number of RCTs for each pairwise comparison. The size of each node is proportional to the number of randomized participants. Metformin (MTN), Pioglitazone (PLZ), Intranasal insulin 20 IU (ITSN20), Intranasal insulin 40 IU (ITSN40), Rosiglitazone 2 mg (RLZ2), Rosiglitazone 4 mg (RLZ4), Rosiglitazone 8 mg (RLZ8), Rosiglitazone 10 mg (RLZ10), Glucagon-like peptide-1 (GLP-1), Dipeptidyl peptidase 4 inhibitor (DPP-4).

Several HDs affect cognitive function, as determined through a comparative analysis of MMSE scores (Figure 2A); the higher the MMSE score, the better the patient's cognitive function. DPP-4 represented a significant improvement over ITSN20, MTN, PLZ and GLP-1 (SMD 2.97, 95% CI (2.24, 3.70); SMD 3.78, 95% CI (2.78, 4.78); SMD 2.83, 95% CI (1.14, 4.52); and SMD 3.33, 95% CI (2.38, 4.28), respectively); a network map is shown in Figure 3A. Compared with ITSN20, MTN had a significant disadvantage [SMD −0.81, 95% CI (−1.57, −0.05)].

The ADAS-Cog score was used to assess memory, language, operating ability and attention; the smaller the difference was, the better the effect of the intervention in improving cognitive function. Analysis and comparison of ADAS-Cog scores (Figure 2B) showed that RLZ8 was significantly lower than MTN and RLZ2 (SMD −1.59, 95% CI (−2.84, −0.34), and SMD −1.40, 95% CI (−2.57, −0.23), respectively]; a network map is shown in Figure 3B.

The ADCS-ADL score is mainly used to assess the ability to carry out common tasks in daily life. The higher the score, the greater the ability to complete these tasks. Analysis of the effect of HDs on the ADCS-ADL scale score showed that ITSN20 and ITSN40 significantly outperformed placebo [SMD 0.02, 95% CI (0.01, 0.03), and SMD 0.04, 95% CI (0.03, 0.05), respectively], and ITSN40 significantly outperformed ITSN20 [SMD 0.02, 95% CI (0.01, 0.03)] (Figure 2C). A network map is shown in Figure 3C.

**Figure 4:**
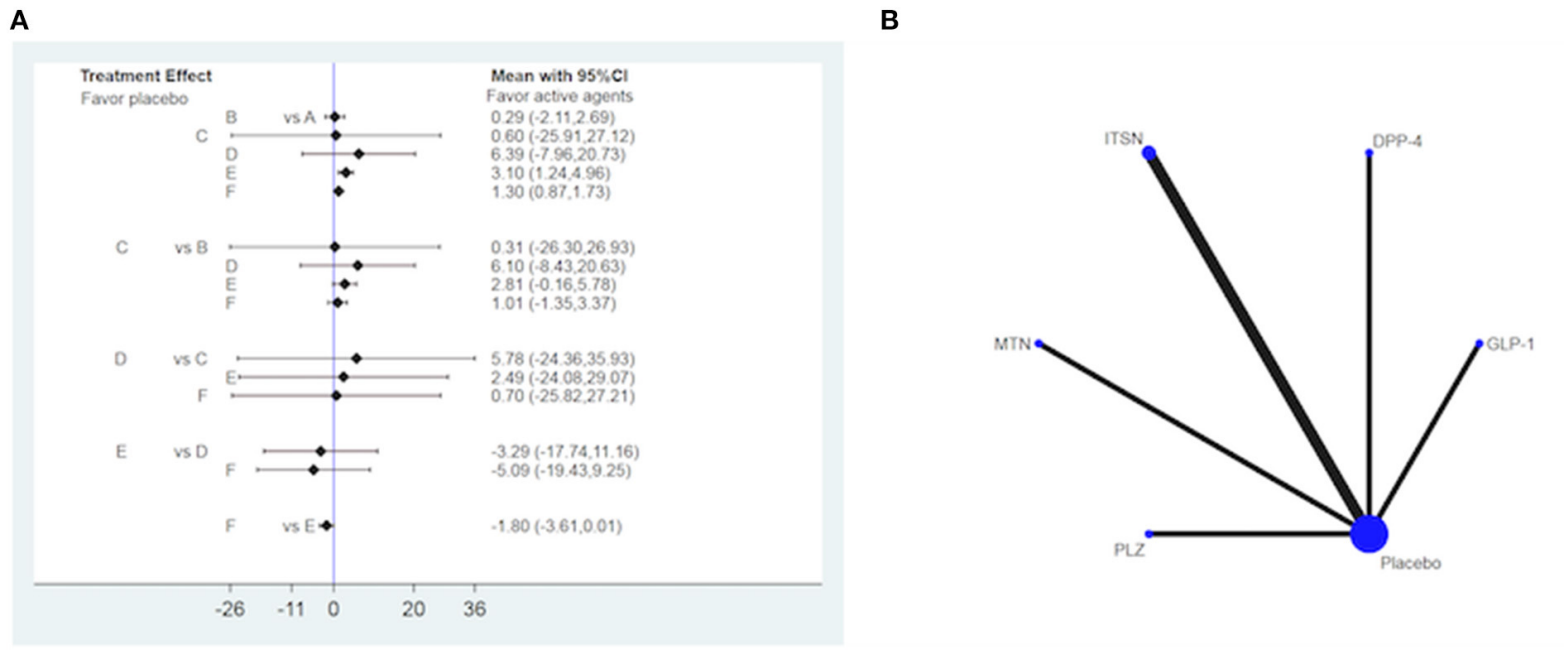
Forest plot **(A)** and network map **(B)** for hypoglycemic drugs (HD) with placebo in improving Aβ42. **(A)** A: placebo, B: intranasal insulin, C: metformin, D: pioglitazone, E: glucagon-like peptide-1, F: dipeptidyl peptidase 4 inhibitor. **(B)** Lines connect the interventions that have been studied in head-to-head comparisons in eligible RCTs. The width of the lines represents the total number of RCTs for each pairwise comparison. The size of each node is proportional to the number of randomized participants. Metformin (MTN), Pioglitazone (PLZ), Intranasal insulin (ITSN), Glucagon-like peptide-1 (GLP-1), Dipeptidyl peptidase 4 inhibitor (DPP-4).

We selected Aβ42 as the blood biochemical index to test AD and MCI. GLP-1 and DPP-4 performed better than placebo [SMD 3.10, 95% CI (1.24, 4.96), and SMD 1.3, 95% CI (0.87, 1.73), respectively] (Figure 4).

### Network meta-analysis of treatment groups

#### Mini-mental state examination

The indirect comparison of the effects of various intervention measures on MCI and AD cognitive function by MMSE, ADAS-Cog and ADCS-ADL shows that DPP-4 and MTN have higher significant effects.

The SUCRA was used to rank the interventions in terms of their effects on MMSE scores. DPP-4 was the best treatment (100%), followed by ITSN (65%), PLZ (62%), GLP-1 (44.8%), MTN (22.6%), and placebo (5.5%). ITSN20 was superior to MTN and placebo [SMD 0.81, 95% CI (0.05, 1.57), (SMD 1.11, 95% CI (0.87, 1.35)], and GLP-1 was superior to placebo [SMD 0.75, 95% CI (0.09, 1.41)] (Table 2).

**Table 2:** Matrix of pairwise comparison among hypoglycemic drugs on MMSE score (shown as mean difference and 95% confidence intervals).

	**DPP-4**	**ITSN20**	**PLZ**	**GLP-1**	**MTN**	**Placebo**
SUCRA (%)	100	65.1	62	44.8	22.6	5.5
DPP-4	0	−2.97 (−3.70, −2.24)	−2.83(−4.52, −1.14)	−3.33 (−4.28, −2.38)	−3.78 (−4.78, −2.78)	−4.08 (−4.77, −3.39)
ITSN20	2.97 (2.24, 3.70)	0	0.14 (−1.42, 1.70)	−0.36 (−1.06, 0.34)	−0.81 (−1.57, −0.05)	−1.11 (−1.35, −0.87)
PLZ	2.83 (1.14, 4.52)	−0.14 (−1.70, 1.42)	0	−0.50 (−2.18, 1.17)	−0.95 (−2.65, 0.75)	−1.25 (−2.79, 0.29)
GLP-1	3.33 (2.38, 4.28)	0.36(−0.34, 1.06)	0.50 (−1.17, 2.18)	0	−0.45 (−1.43, 0.53)	−0.75 (−1.41, −0.09)
MTN	3.78 (2.78, 4.78)	0.81 (0.05, 1.57)	0.95 (−0.75, 2.65)	0.45 (−0.53, 1.43)	0	−0.30 (−1.02, 0.42)
Placebo	4.08 (3.39, 4.77)	1.11 (0.87, 1.35)	1.25 (−0.29, 2.79)	0.75 (0.09, 1.41)	0.30 (−0.42, 1.02)	0

SUCRA, Surface under the cumulative ranking curve; ITSN20, Intranasal insulin 20IU; MTN, Metformin; PLZ, Pioglitazone;, RLZ2, Rosiglitazone 2 mg; RLZ4, Rosiglitazone 4 mg; RLZ8, Rosiglitazone 8mg; RLZ10, Rosiglitazone 10 mg; GLP-1, Glucagon-like Peptide-1; DPP-4, Dipeptidyl Peptidase 4 Inhibitor; MMSE, Mini-mental State Examination.

#### ADAS-Cog score

As determined by the SUCRA, MTN was the best treatment to improve ADAS-Cog scores. The treatments, from most to least effective, were as follows: MTN (86.6%), placebo (82.4%), RLZ2 (79.6%), PLZ (61.6%), RLZ10 (52.2%), GLP-1 (47.6%), ITSN40 (34.8%), RLZ4 (31.8%), RLZ8 (12.9%), ITSN20 (10.5%). MTN significantly outperformed RLZ8 [SMD 1.59, 95% CI (0.34, 2.84)]. RLZ10, ITSN40, RLZ4, and ITSN20 were significantly worse than placebo [SMD 3.02, 95% CI (1.86, 4.17); SMD 1.88, 95% CI (1.66, 3.09); SMD 1.40, 95% CI (0.23, 2.57); and SMD 0.92, 95% CI (0.08, 1.77), respectively]. RLZ2 was significantly better than RLZ8 [SMD 1.40, 95% CI (0.23, 2.57)]. ITSN20 was better than RLZ8 [SMD 1.55, 95% CI (0.21, 2.90)] (Table 3).

**Table 3:** Matrix of pairwise comparison among hypoglycemic drugs on ADAS-Cog score (shown as mean difference and 95% confidence intervals).

	**MTN**	**Placebo**	**RLZ2**	**PLZ**	**RLZ10**	**GLP-1**	**ITSN40**	**RLZ4**	**RLZ8**	**ITSN20**
SUCRA (%)	86.6	82.4	79.6	61.6	52.2	47.6	34.8	31.8	12.9	10.5
MTN	0	−0.20 (−1.11, 0.72)	−0.19 (−1.41, 1.04)	−0.55 (−1.74, 0.64)	−0.75 (−2.00, 0.49)	−0.82 (−2.17, 0.53)	−1.08 (−2.22, 0.06)	−1.12 (−2.37, 0.12)	−1.59 (−2.84, −0.34)	−0.03 (-1.42,1.35)
Placebo	0.20 (−0.72, 1.11)	0	0.36 (−0.81, 1.53)	−0.35 (−1.11, 0.40)	−3.02 (−4.17, −1.86)	−0.62 (−1.62, 0.37)	−1.88 (−3.09, −0.66)	−1.40 (−2.57, −0.23)	0.01 (−0.80, 0.82)	−0.92 (-1.77,-0.08)
RLZ2	0.19 (−1.04, 1.41)	−0.36 (−1.53, 0.81)	0	−0.37 (−1.47, 0.74)	−0.57 (−1.74, 0.60)	−0.63 (−1.92, 0.65)	−0.90 (−1.95, 0.16)	−0.94 (−2.11, 0.23)	−1.40 (−2.57, −0.23)	0.15 (-1.17,1.47)
PLZ	0.55 (−0.64, 1.74)	0.35 (−0.40, 1.11)	0.37 (−0.74, 1.47)	0	−0.20 (−1.33, 0.93)	−0.27 (−1.52, 0.98)	−0.53 (−1.55, 0.48)	−0.57 (−1.70, 0.56)	−1.04 (−2.17, 0.10)	0.52 (-0.77,1.80)
RLZ10	0.75 (−0.49, 2.00)	3.02 (1.86, 4.17)	0.57 (−0.60, 1.74)	0.20 (−0.93, 1.33)	0	−0.07 (−1.37, 1.24)	−0.33 (−1.41, 0.75)	−0.37 (−1.21, 0.47)	−0.83 (−2.03, 0.36)	0.72 (-0.62,2.06)
GLP-1	0.82 (−0.53, 2.17)	0.62 (−0.37, 1.62)	0.63 (−0.65, 1.92)	0.27 (−0.98, 1.52)	0.07 (−1.24, 1.37)	0	−0.26 (−1.47, 0.94)	−0.30 (−1.61, 1.00)	−0.77 (−2.07, 0.54)	0.78 (-0.66,2.23)
ITSN40	1.08 (−0.06, 2.22)	1.88 (0.66, 3.09)	0.90 (−0.16, 1.95)	0.53 (−0.48, 1.55)	0.33 (−0.75, 1.41)	0.26 (−0.94, 1.47)	0	−0.04 (−1.12, 1.04)	−0.50 (−1.59, 0.58)	1.05 (-0.20,2.29)
RLZ4	1.12 (−0.12, 2.37)	1.40 (0.23, 2.57)	0.94 (−0.23, 2.11)	0.57 (−0.56, 1.70)	0.37 (−0.47, 1.21)	0.30 (−1.00, 1.61)	0.04 (−1.04, 1.12)	0	−0.47 (−1.66, 0.73)	1.09 (-0.25,2.43)
RLZ8	1.59 (0.34, 2.84)	−0.01 (−0.82, 0.80)	1.40 (0.23, 2.57)	1.04 (−0.10, 2.17)	0.83 (−0.36, 2.03)	0.77 (−0.54, 2.07)	0.50 (−0.58, 1.59)	0.47 (−0.73, 1.66)	0	1.55 (0.21,2.90)
ITSN20	0.03 (−1.35, 1.42)	0.92 (0.08, 1.77)	−0.15 (−1.47, 1.17)	−0.52 (−1.80, 0.77)	−0.72 (−2.06, 0.62)	−0.78 (−2.23, 0.66)	−1.05 (−2.29, 0.20)	−1.09 (−2.43, 0.25)	−1.55 (−2.90, −0.21)	0

SUCRA, Surface under the cumulative ranking curve; ITSN20, Intranasal insulin 20IU; ITSN40, Intranasal insulin 40IU; MTN, Metformin; PLZ, Pioglitazone; RLZ2, Rosiglitazone 2 mg; RLZ4, Rosiglitazone 4 mg; RLZ8, Rosiglitazone 8 mg; RLZ10, Rosiglitazone 10 mg; GLP-1, Glucagon-like Peptide-1; ADAS-Cog score, Alzheimer's Disease Assessment Scale-Cognitive section.

#### ADCS-ADL scale score

By SUCRA ranking analysis, placebo (100%) was superior to ITSN20 (50.1%) and ITSN40 (0.1%) [SMD −0.02, 95% CI (−0.03, −0.01), and SMD −0.04, 95% CI (−0.05, −0.03), respectively)](Table 4).

**Table 4:** Matrix of pairwise comparison among hypoglycemic drugs on ADCS-ADL scale score (shown as mean difference and 95% confidence intervals).

	**Placebo**	**ITSN20**	**ITSN40**
SUCRA (%)	99.8	50.1	0.1
Placebo	0	0.02 (0.01,0.03)	0.04 (0.03,0.05)
ITSN20	−0.02 (−0.03, −0.01)	0	0.02 (0.01,0.03)
ITSN40	−0.04 (−0.05, −0.03)	−0.02 (−0.03, −0.01)	0

SUCRA, Surface under the cumulative ranking curve; ITSN20, Intranasal insulin 20IU; ITSN40, Intranasal insulin 40IU; ADCS-ADL, Alzheimer's Disease Cooperative Study-Activities of Daily Living Subscal.

#### Adverse events

Of the 16 studies included in the network meta-analysis, nine mentioned the occurrence of adverse events. Among them, oedema, rhinitis, dizziness, and diarrhoea were the most common (Table 5).

**Table 5:** Adverse events included in the literature.

**Adverse events**	**Control(n)**	**Intervention(n)**
Rhinitis	Placebo 3	ITSN 17
Headache/Dizziness	Placebo 22	ITSN 19
		RLZ 35
Musculoskeletal Injury/Pain	Placebo 10	ITSN 12
		RLZ 25
Nose bleed/ Nasal irritation	Placebo 26	ITSN 68
Gastointestinal Symptoms/Diarrhea	Placebo 32	ITSN 62
Fall	Placebo 31	ITSN 5
		RLZ 31
Rash	Placebo 2	ITSN 3
Upper respiratory tract infection	Placebo 2	ITSN 3
Respiratory/sinus symptoms	Placebo 3	ITSN 7
Dental	Placebo 1	ITSN 3
Infection	Placebo 0	ITSN 2
Cardiovascular	Placebo 0	ITSN 2
Nausea	Placebo 4	ITSN 5
Cold symptoms	Placebo 14	PLZ 14
Glucose level low/asymptomatic	Placebo 4	PLZ 4
Swelling/edema of leg/ Peripheral edema	Placebo 11	PLZ 7
		RLZ 106
Anemia	Placebo 4	PLZ 3
		RLZ 26
Fatigue	Placebo 5	PLZ 3
Increased confusion	Placebo 5	PLZ 3
Insomnia	Placebo 2	RLZ 5
Other (stroke, breast cancer, weakness, ophthalmic, hypoglycemia, hematological, hearing loss, endocrine)	Placebo 18	ITSN 70

MTN, Metformin; ITSN, Intranasal insulin; PLZ, Pioglitazone; RLZ, Rosiglitazone.

#### Sensitivity and publication bias

Sensitivity analysis showed that any single study or cluster study with certain characteristics had little effect on the change in SMD and its corresponding 95% CI. Significant publication bias was not reported by Egger's regression test or Begg's adjusted rank correlation test.

## Discussion

According to current studies, most HDs have a considerable positive effect on the cognitive function of patients with AD and MCI. Only GLP-1 and DPP-4 have an inhibitory effect on the accumulation of Aβ42, and the impact on other biomarker indicators still needs more research. For the three different scoring standards of cognitive function, the efficacy of various HDs was different. In our study, for MMSE, DPP-4 was better than other HDs. For ADAS-Cog scores, 8 mg RLZ was better than MTN and 2 mg RLZ. For the ADCS-ADL scale, the curative effect of ITSN 40 IU was better than that of ITSN 20 IU.

Previous studies have shown that PLZ 15–30 mg, an HD, has an extraordinary promoting cognitive effect for MCI/AD (36). However, many measurement standards are not insufficient to evaluate how HD improves cognitive function. To the best of our knowledge, our network meta-analysis was the first to compare six types of HD-ameliorated MCI/AD and analysed the different criteria for evaluating cognitive function by pairwise comparison.

Both hippocampus and connected limbic brain structures in the brain are memory-forming regions with a high density of insulin receptors. Insulin signals contribute to neuronal plasticity and regulate the cognitive function and memory function of the brain (37). The intranasal pathway was capable of delivering insulin to the central nervous system with relatively no systemic absorption and associated peripheral side effects. Intranasal insulin (ITSN) rapidly accumulates in the cerebrospinal fluid and is efficiently transported to the brain (38). Studies have shown (39, 40) that insulin is involved in synaptic plasticity in the brain, i.e., long-term potentiation (LTP) and long-term depression (LTD), and the establishment of the hippocampal memory locus depends on LTP and LTD. Insulin regulates LTD by inducing internalization of the glutamate AMPA receptor. ITSN enhances long-term declarative memory without hypoglycaemic side effects and enhances functional connectivity between the prefrontal cortex and the hippocampus in diabetes mellitus type 2 (37, 41). Previous studies (42, 43) have shown that ITSN40 can improve memory and cognitive function, with few negative effects, which was consistent with our analysis that ITSN40 was more effective and safer than ITSN 20.

Metformin (MTN), a HD commonly used in most health guidelines, can cross the blood–brain barrier (BBB) and is involved in cognitive improvement (44). Previous studies (45, 46) showed that MTN not only significantly reduced the hyperphosphorylation of tau and APPc99 but also improved the learning and memory performance of SAMP8 and APP/PS1 transgenic mice based on Morris water maze and Y-maze results. MTN can significantly reduce β-secretase1 (BACE1) protein expression and activity, reducing BACE1 cleavage products and Aβ (47). The AMPK pathway in human neural stem cells (NCS) can be activated by MTN, which is considered a potential therapeutic target of AD (48). Saliu et al. (49) confirmed that MTN at a dose of 500 mg/kg can significantly reduce AChE activity in the brains of streptozotocin-induced diabetic rats. MTN has many ways to improve neural cells and can effectively improve the cognitive impairment of patients with type 2 diabetes clinically. In our study, the metformin group had lower (better) ADAS-Cog scores (*p* = 0.02), but other biological indicators were not mentioned.

Pioglitazone (PLZ) and rosiglitazone (RLZ) are insulin sensitizers of thiazolidinedione nuclear peroxisome proliferator activated receptor γ (PPARγ) agonists. PLZ was found to reduce glial inflammation and Aβ levels in the brains of transgenic mice, enhancing microglial uptake of Aβ in a PPARγ-dependent manner (50, 51). RLZ can reduce the expression of inflammatory cytokines and improve cognitive function by inhibiting the activation of NF-κB signalling in the hippocampus (52). RLZ can regulate several processes related to AD, such as reducing tau and amyloid pathology and inhibiting inflammation (53). For the treatment of MCI/AD with RLZ, the selection of dose was particularly important. In our analysis, RLZ was divided into 2, 4, 8, and 10 mg, and the dose was not directly proportional to the effect. In terms of the ADAS-Cog score, 2 mg had a better efficacy than 10 mg, but 10 mg was better than 4 and 8 mg.

Glucagon-like peptide-1 (GLP-1) is derived from the glucagon gene. It is produced in the central nervous system (CNS), mainly in the brain stem, and then transported to a large number of areas of the central nervous system (54). GLP-1 enhances central insulin resistance, promotes the growth of synapses and neurons, and prevents oxidative damage (55). In an AD mouse model, GLP-1 agonists reduce the level of AD pathological markers, including oligomer Aβ and Aβ plaque load, reduce the activation of microglia, and improve memory behaviour (56). In our study, the GLP group had better performance on all cognitive tests, which included the total learning (*p* = 0.039), animal naming test (*p* = 0.025), and MMSE (*p* = 0.001). For blood glucose indicators, the concentrations of FBG and HbA1c in the GLP group and the control group decreased significantly, but there was no significant difference between the two groups.

Dipeptidyl peptidase-4 (DPP-4) inhibitors exist in blood plasma and cerebrospinal fluid and exert their multifunctional functions through the influence of various signalling pathways, inducing and regulating inflammatory and immune processes (57). DPP-4 inhibitors have recently been shown to have important neuroprotective effects, reversing the pathophysiological processes of AD and improving the cognitive abilities of AD animal models and patients (58). It has also been suggested (59) that DPP-4 inhibitors tend to improve MCI. The number of related studies involving DPP-4 inhibitors in our included literature was small, so further studies are needed. Antonio et al. (60) used DPP-4+metformin as an intervention measure in their study, and the results showed that the combination of two HDs had a better effect in improving the MMSE score than metformin alone (*p* < 0.001). Through a further search of the database, we found that there were few clinical studies of the combined application of two HDs in the treatment of MCI/AD. Therefore, large sample and multi-centre RCTs are needed. Clinically, the combination of HD can be used to improve the symptoms of MCI/AD patients, especially those with type 2 diabetes.

### Limitations

First, the sample size included in the network meta-analysis was relatively small, and there were few studies with comparable original outcome indicators. Second, there were few biological indicators related to MCI/AD, and the analysis was not thorough enough. Finally, the observation time, drug dose and duration of medication of the RCTs included in our study were different. It is not ruled out that some unused databases have literature matching the inclusion criteria, which may affect the results.

## Conclusion

The study provides a theoretical basis for the effect of different doses of HDs on MCI/AD cognitive function. Such large-scale, multi-centre and repetitive studies are necessary, and the specific mechanisms of different HDs to improve MCI/AD of different degrees need to be further studied.

## Data availability statement

The original contributions presented in the study are included in the article/Supplementary material, further inquiries can be directed to the corresponding authors.

## Author contributions

X-CW and C-LC: completed the data analysis. X-CW, C-LC, and H-CL: wrote and revised the manuscript. S-JZ and C-LC: conceived and designed the study. X-CW: received financial support. Y-FC and S-JQ: reviewed the full text and approved the final manuscript submitted. All authors contributed to the article and approved the submitted version.

## Funding

This work was supported by Zhaoqing University Excellent Young Teachers' scientific research ability improvement project (No. YQ202102) and Science and Technology Innovation Guidance Project of Zhaoqing Science and Technology Bureau (No. 2022040311001).

## Conflict of interest

The authors declare that the research was conducted in the absence of any commercial or financial relationships that could be construed as a potential conflict of interest.

## Publisher's note

All claims expressed in this article are solely those of the authors and do not necessarily represent those of their affiliated organizations, or those of the publisher, the editors and the reviewers. Any product that may be evaluated in this article, or claim that may be made by its manufacturer, is not guaranteed or endorsed by the publisher.
